# Correction: Working life sequences over the life course among 9269 women and men in Sweden; A prospective cohort study

**DOI:** 10.1371/journal.pone.0319833

**Published:** 2025-03-05

**Authors:** Katalin Gémes, Katriina Heikkilä, Kristina Alexanderson, Kristin Farrants, Ellenor Mittendorfer-Rutz, Marianna Virtanen

S5 Table is a duplicate of S6 Table. Please view the correct S5 Table below.

## Supporting information

S5 Table Measures of cluster partitions quality for different numbers of sequence clusters. (DOCX)
