# Supplementary figures and images for: Working life sequences over the life course among 9269 women and men in Sweden; a prospective cohort study
Source: PLoS One. 2023 Feb 15;18(2):e0281056. doi: 10.1371/journal.pone.0281056 (PMC9931102; doi:10.1371/journal.pone.0281056)

## Flowchart of the selection of study participants

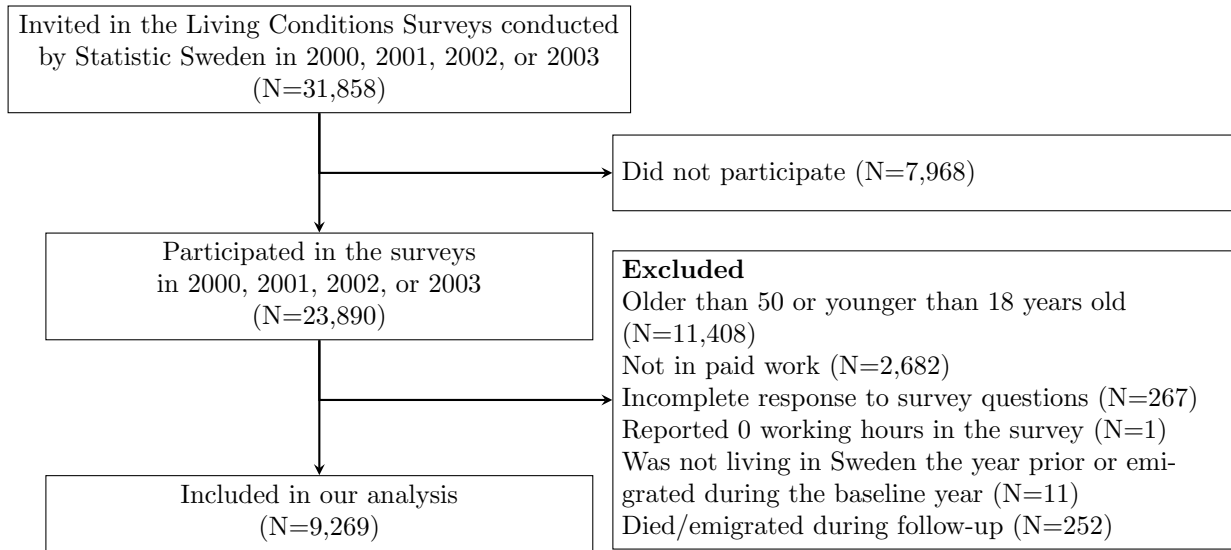

Supplement: S1 Fig — (PDF) [file pone.0281056.s001.pdf]

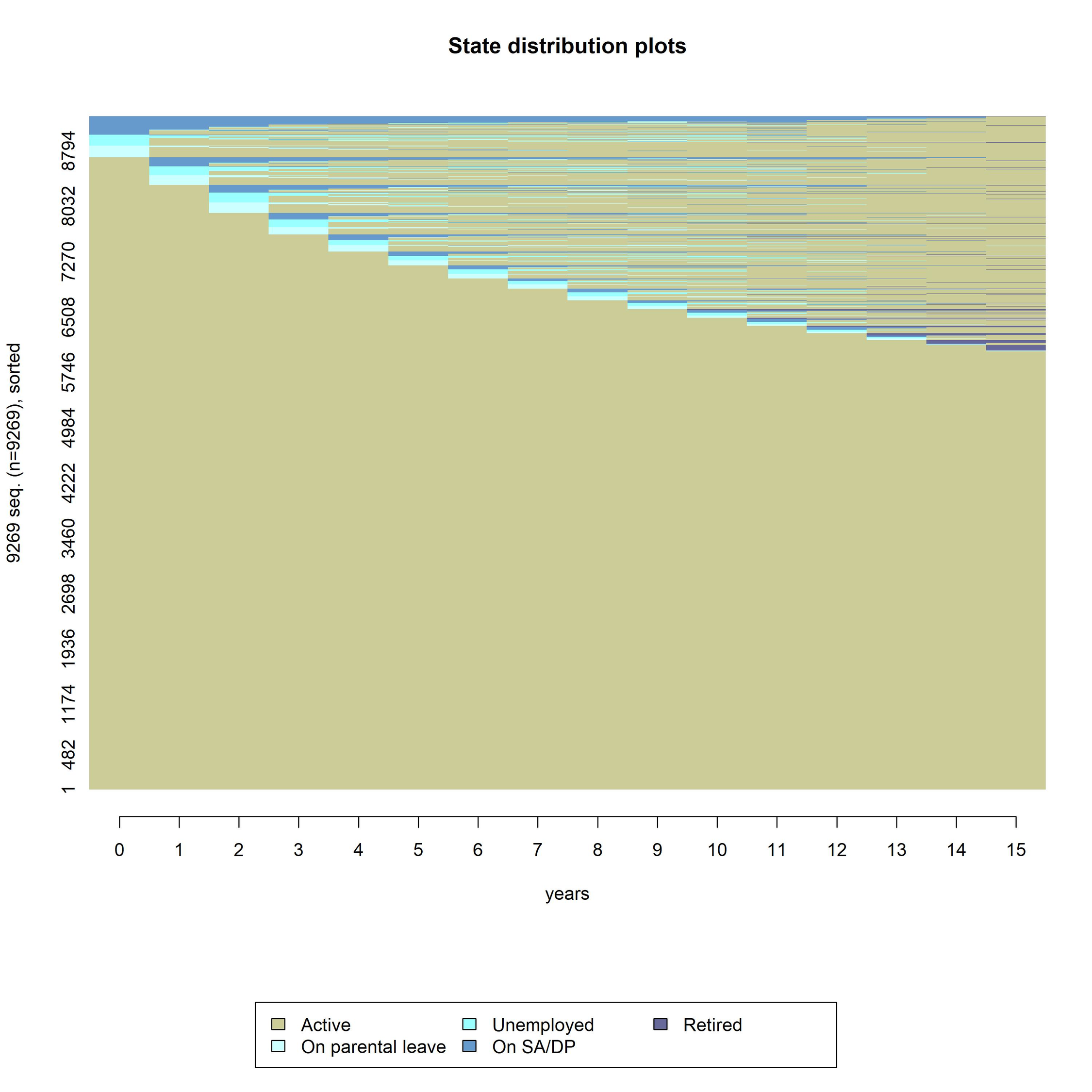

Supplement: S2 Fig — (TIF) [file pone.0281056.s002.tif]

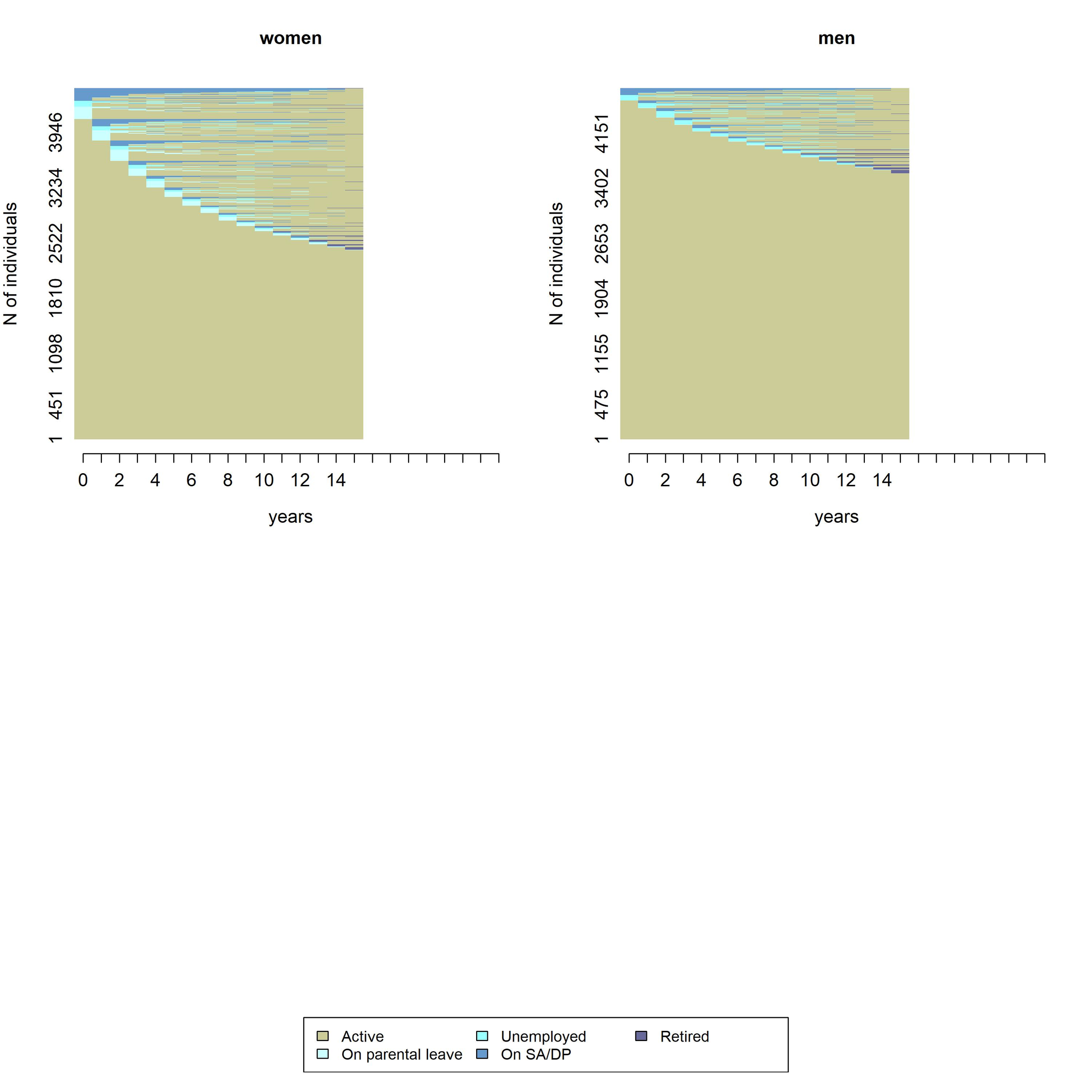

Supplement: S3 Fig — (TIF) [file pone.0281056.s003.tif]

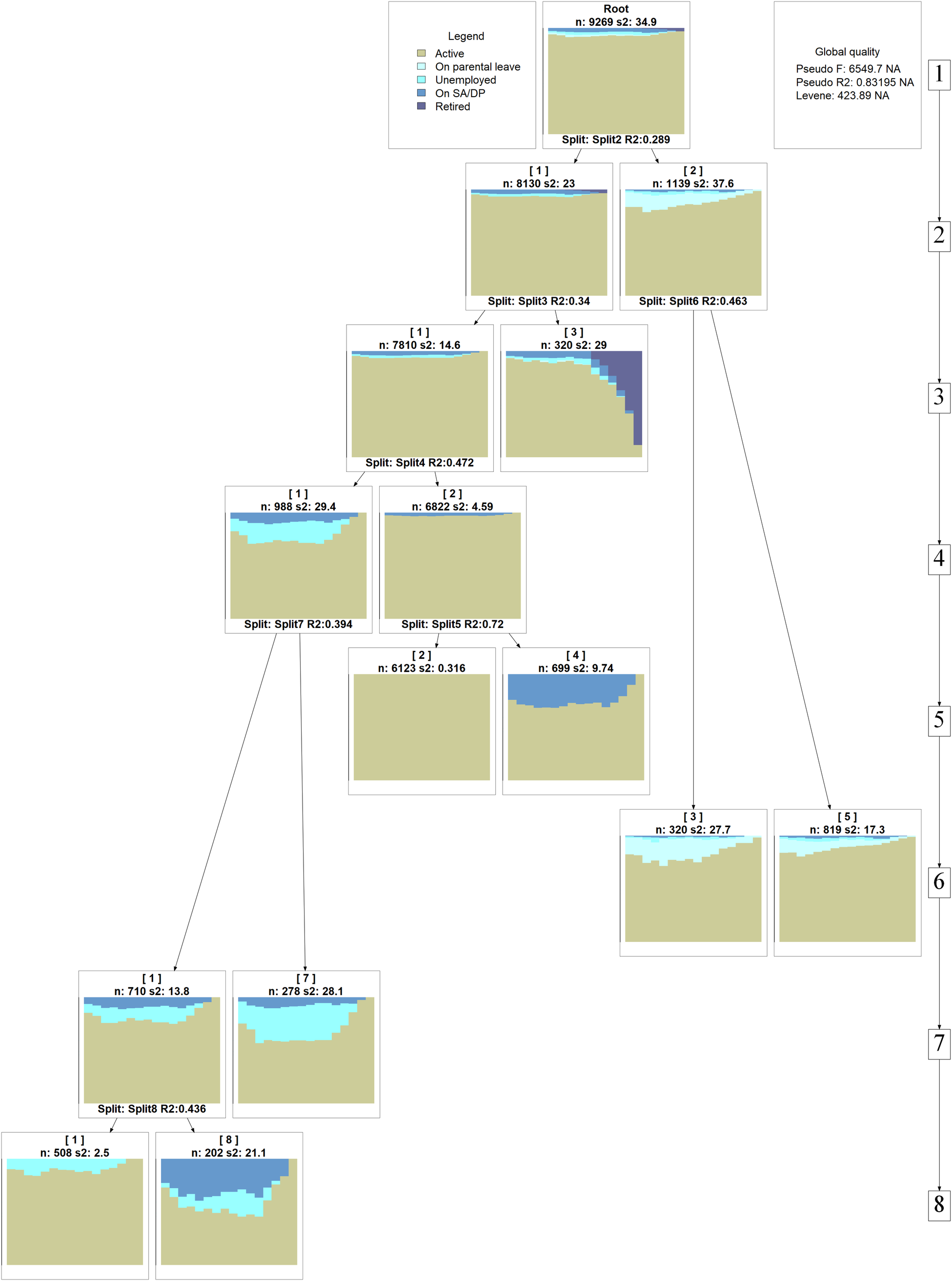

Supplement: S4 Fig — (TIF) [file pone.0281056.s004.tif]

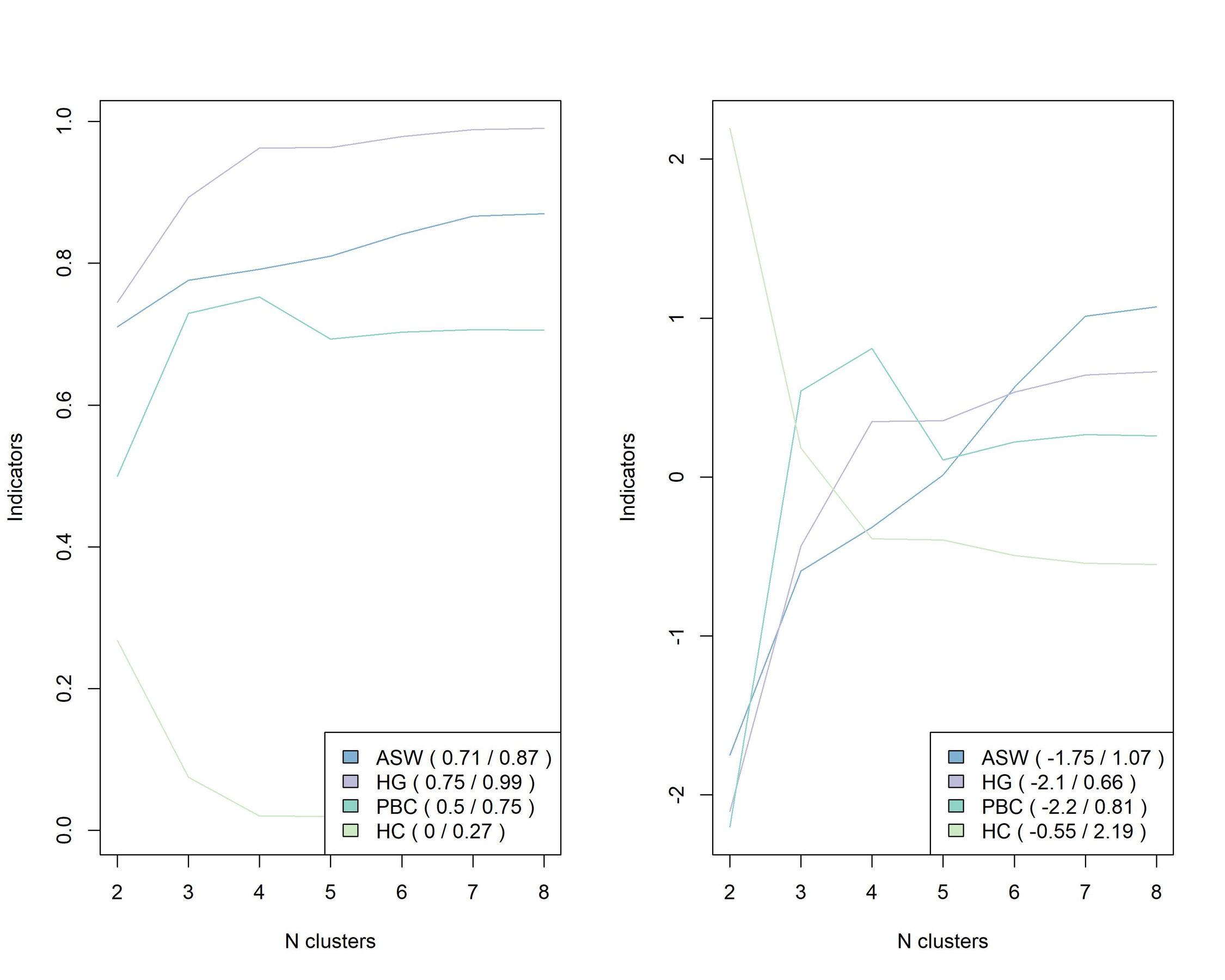

Supplement: S5 Fig — ASW: Average Silhouette Width HG: Hubert’s Gamma, PBC: Point Biserial Correlation HC: Hubert’s C. (TIF) [file pone.0281056.s005.tif]

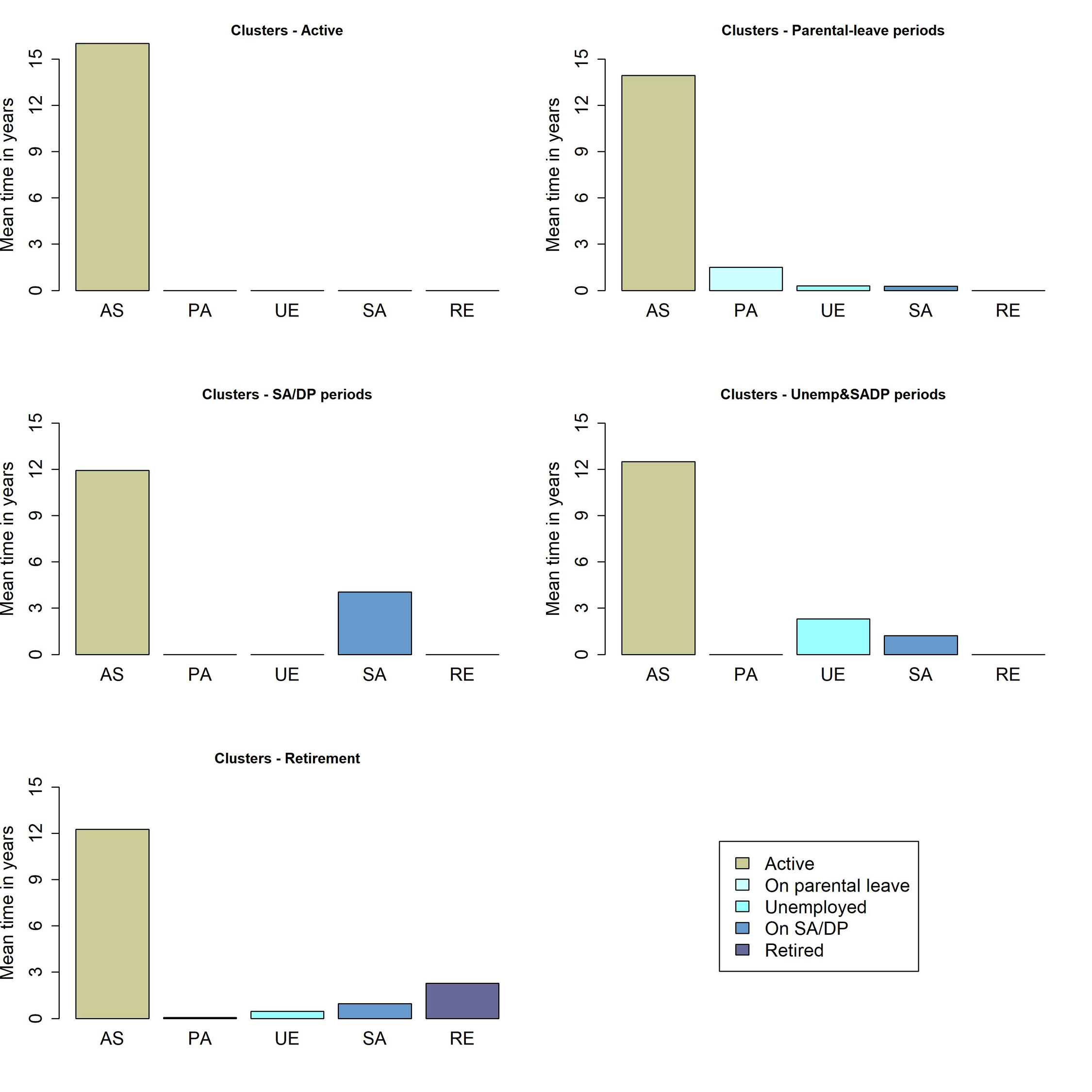

Supplement: S6 Fig — (TIF) [file pone.0281056.s006.tif]

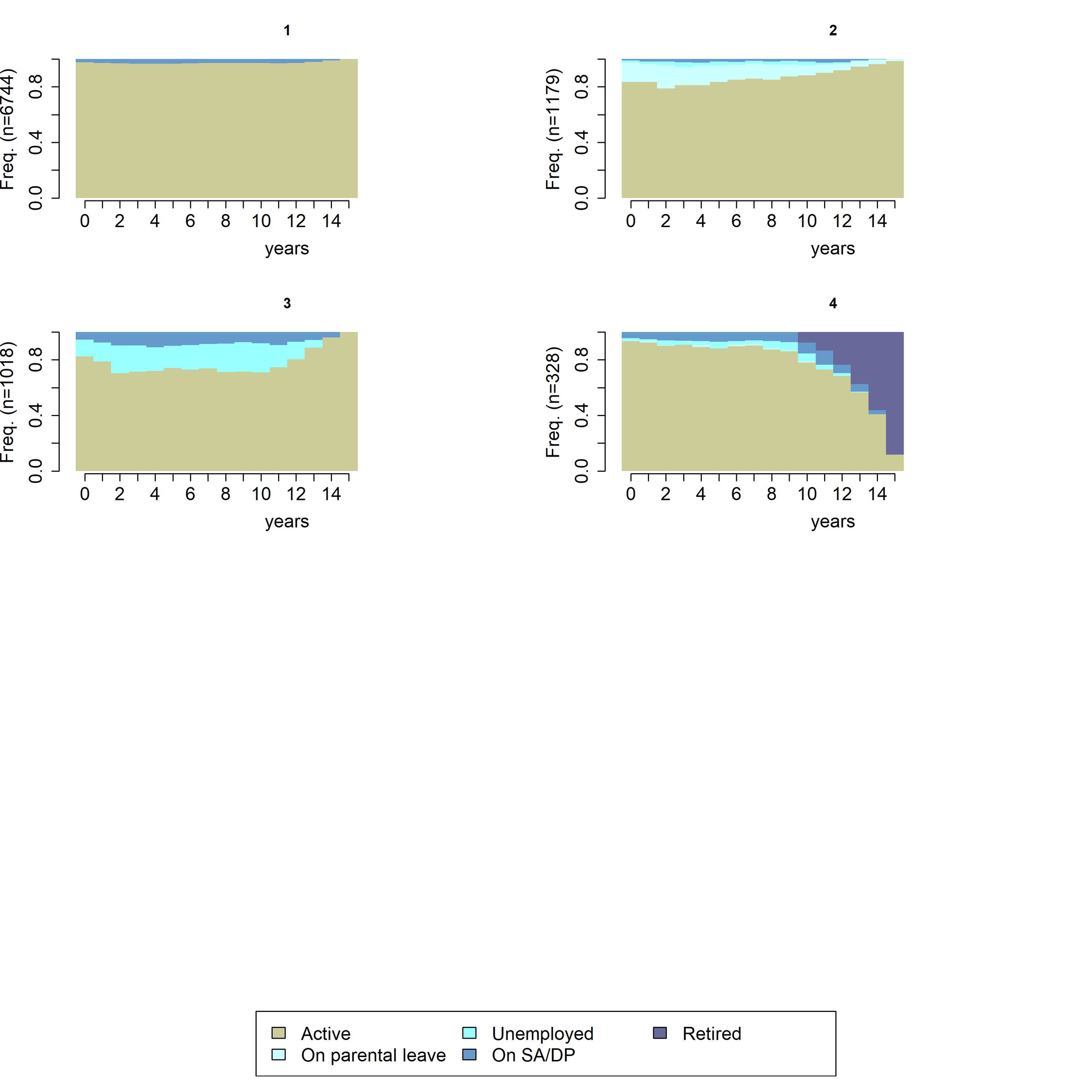

Supplement: S7 Fig — (TIF) [file pone.0281056.s007.tif]

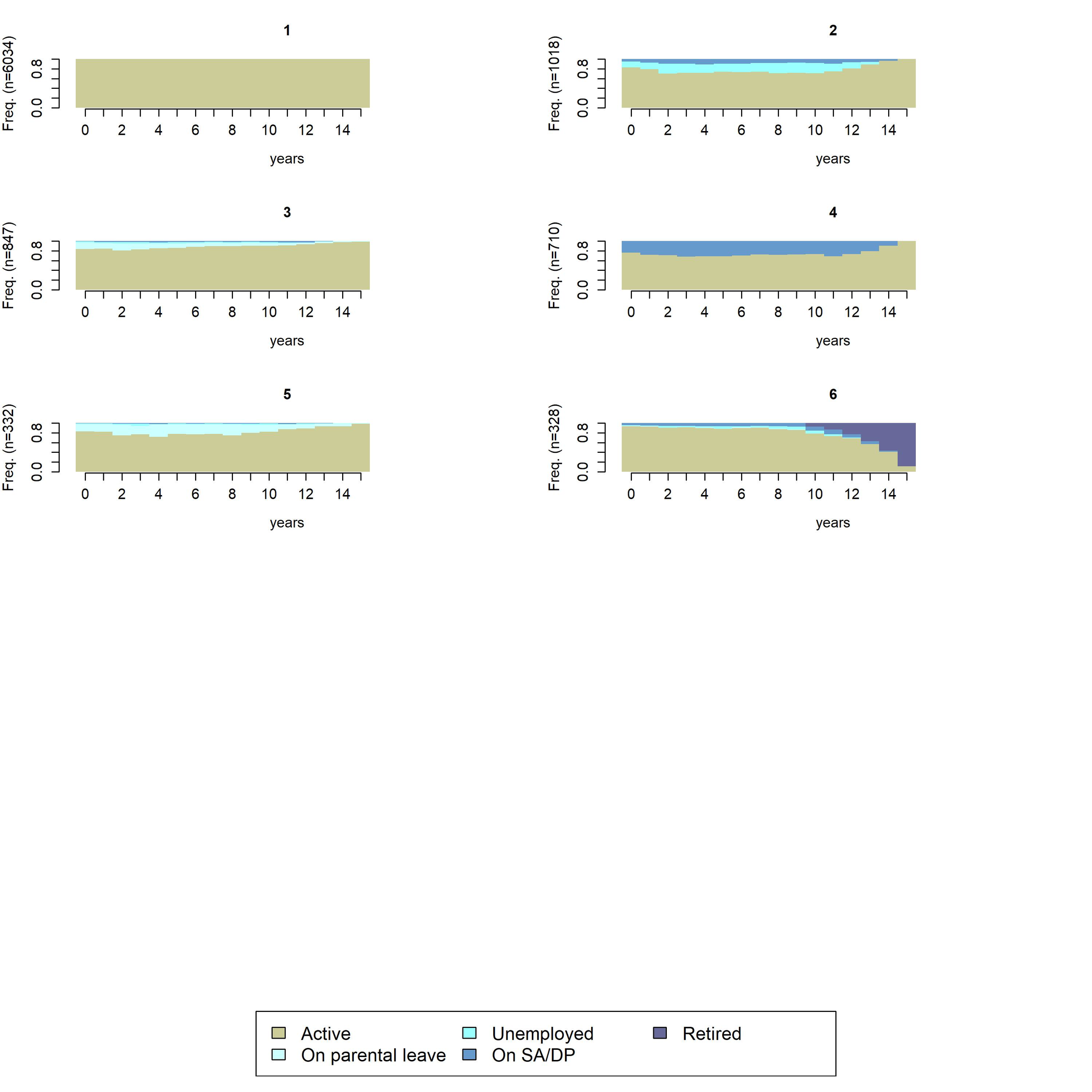

Supplement: S8 Fig — (TIF) [file pone.0281056.s008.tif]

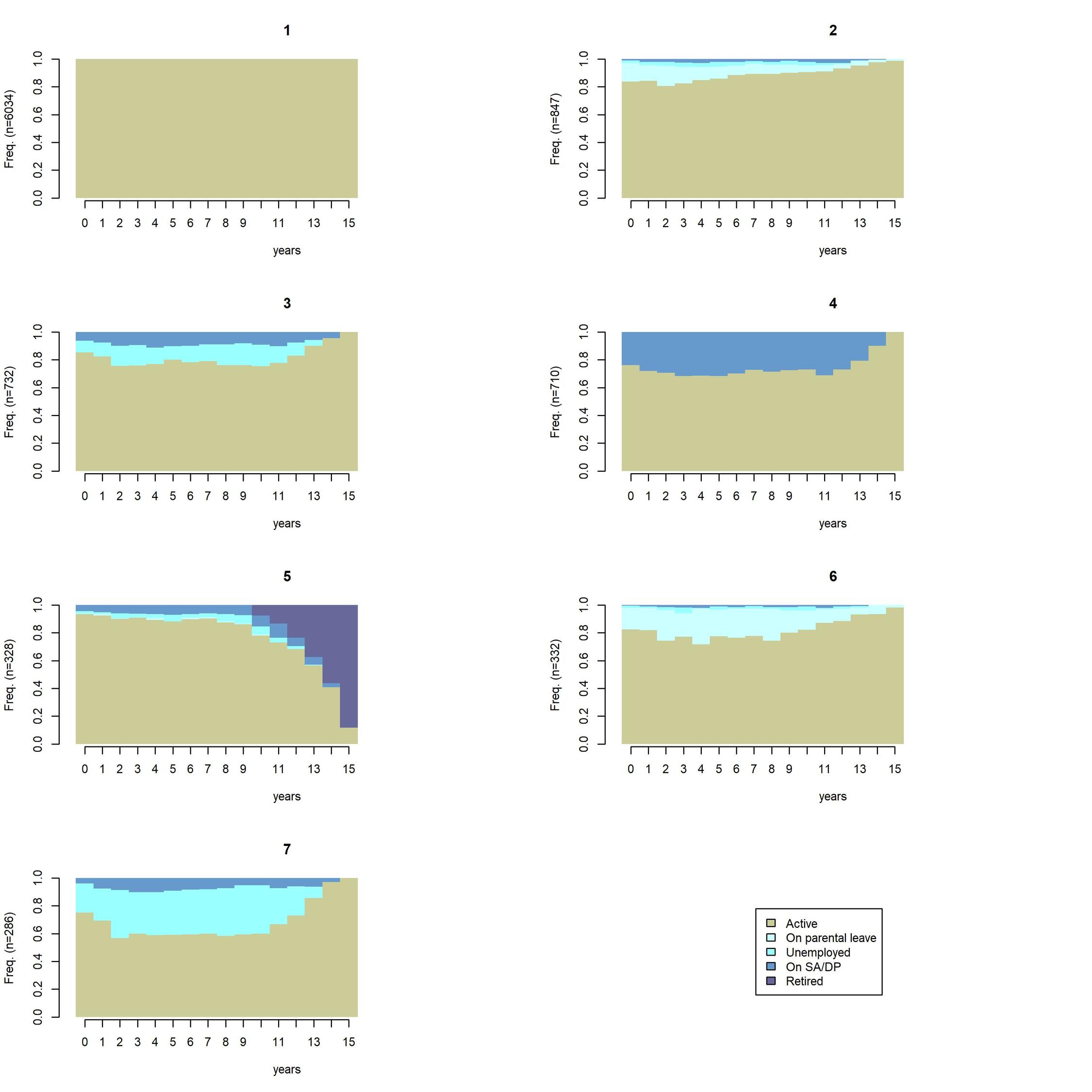

Supplement: S9 Fig — (TIF) [file pone.0281056.s009.tif]

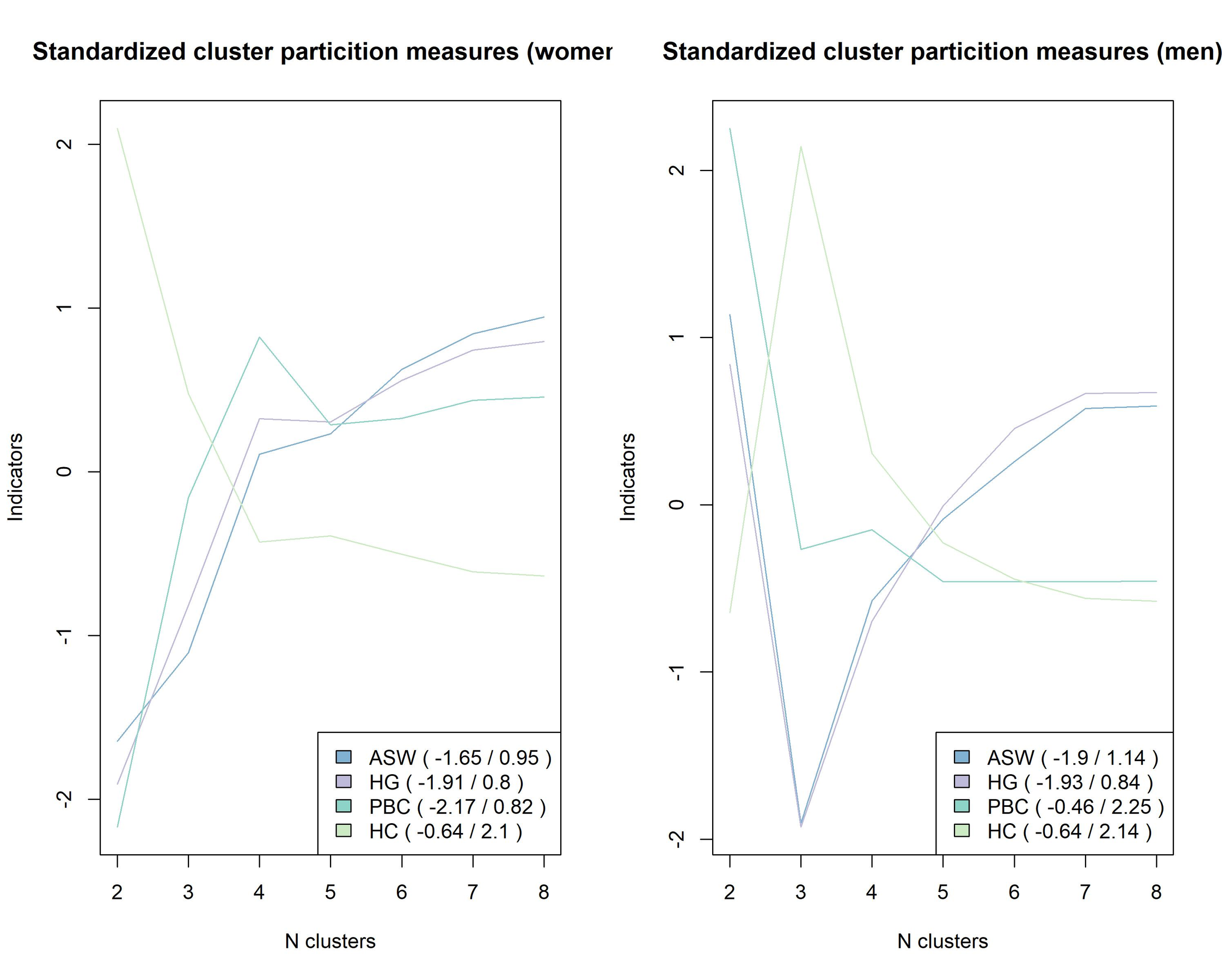

Supplement: S10 Fig — (TIF) [file pone.0281056.s010.tif]

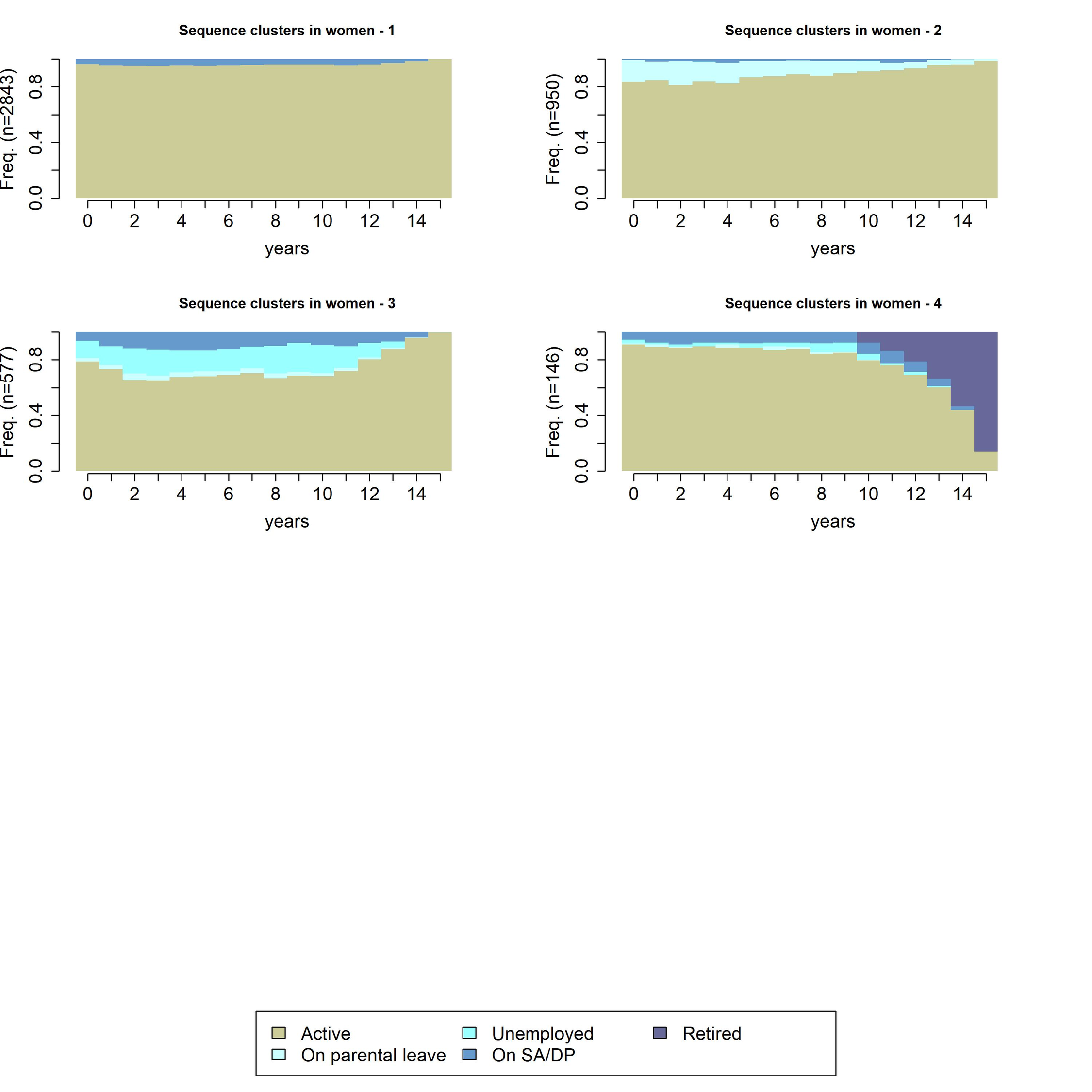

Supplement: S11 Fig — (TIF) [file pone.0281056.s011.tif]

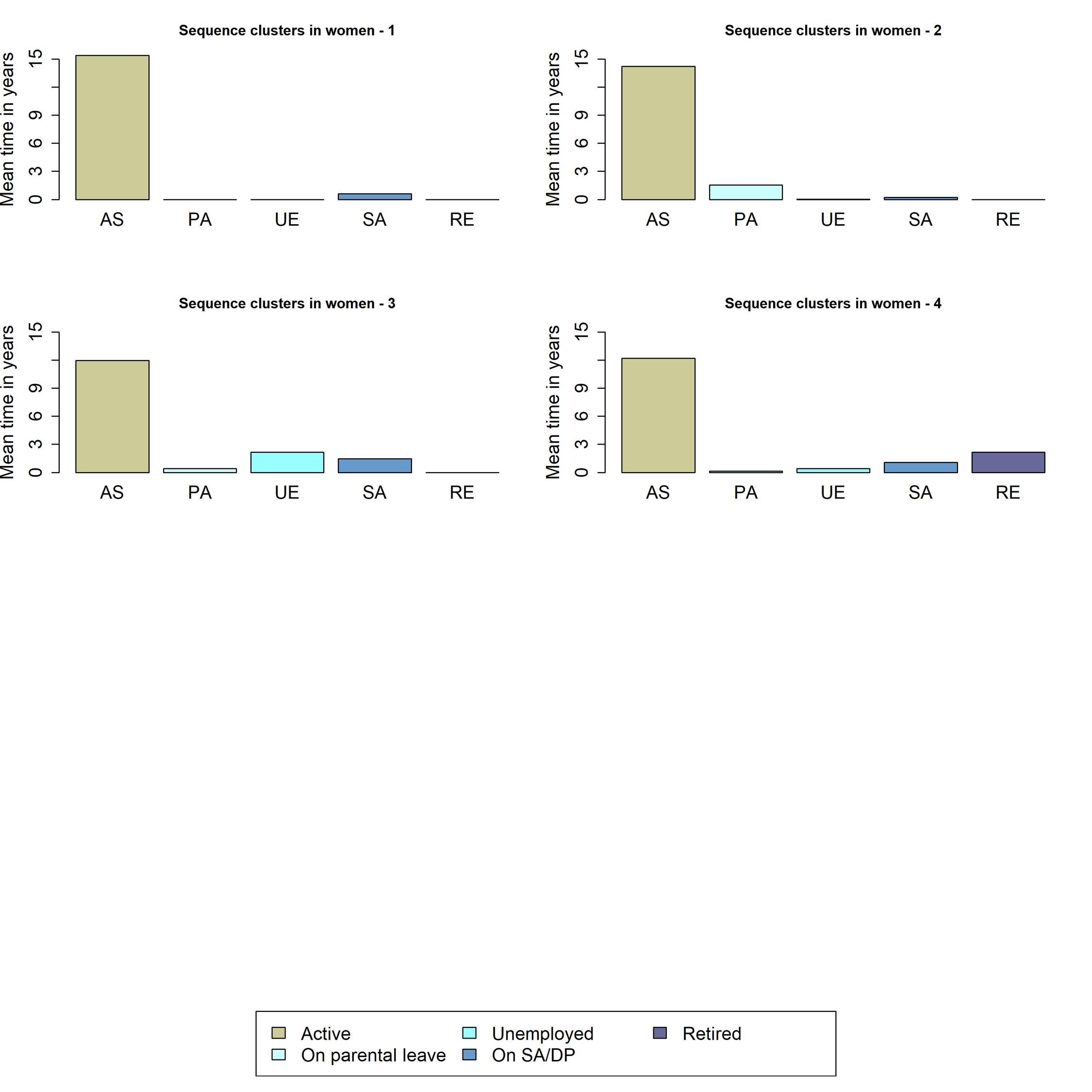

Supplement: S12 Fig — (TIF) [file pone.0281056.s012.tif]

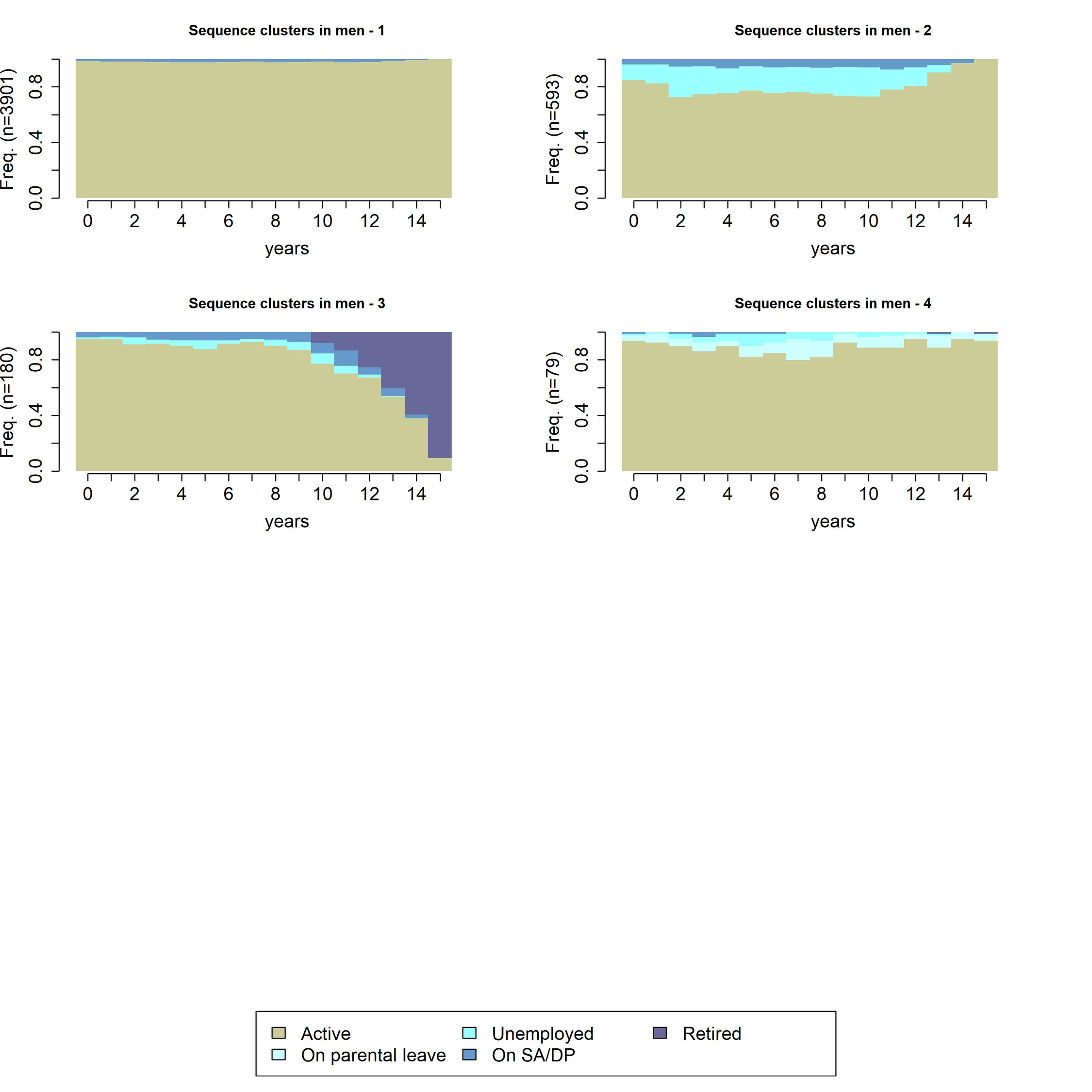

Supplement: S13 Fig — (TIF) [file pone.0281056.s013.tif]

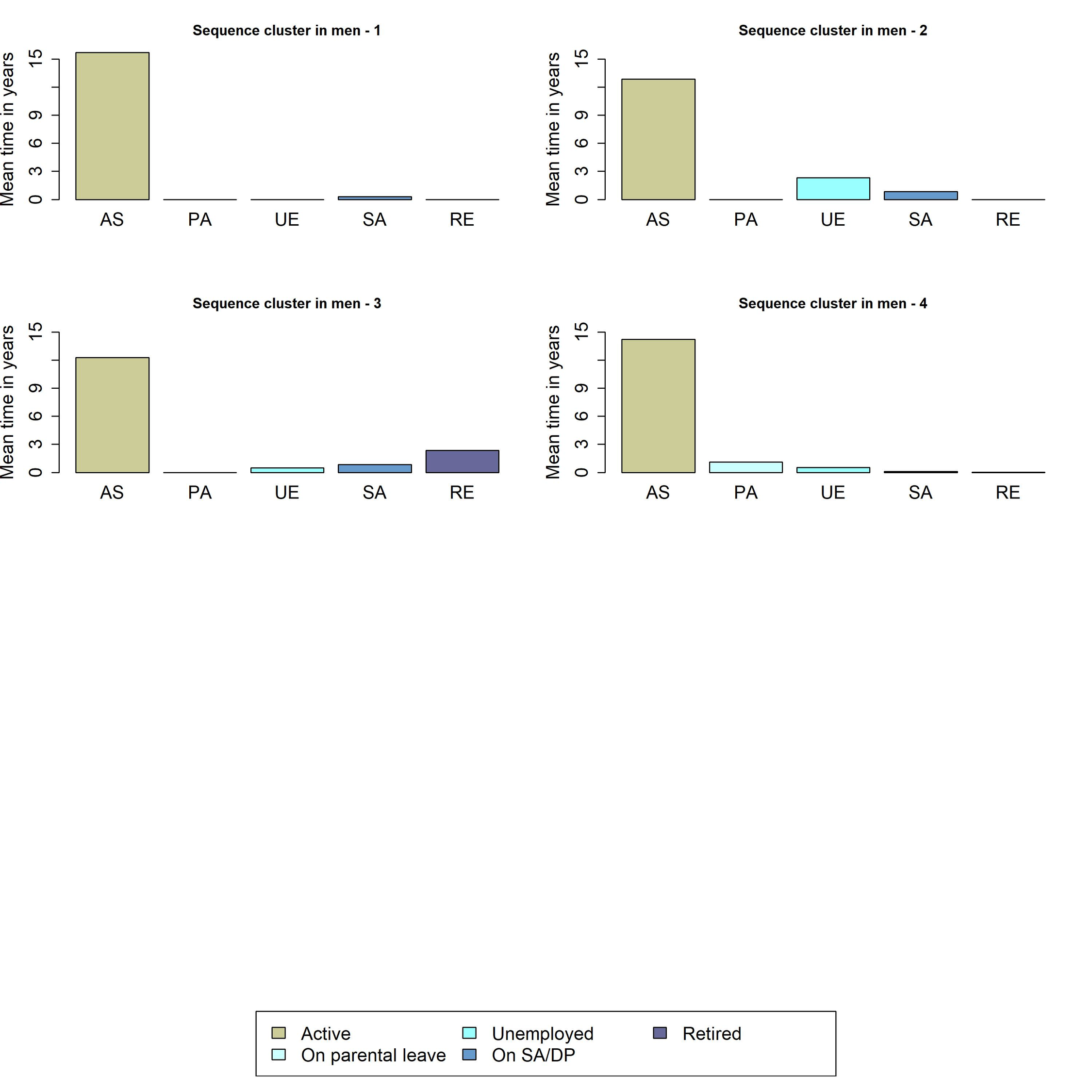

Supplement: S14 Fig — (TIF) [file pone.0281056.s014.tif]
