# Supplementary material for: Working life sequences over the life course among 9269 women and men in Sweden; a prospective cohort study
Source: PLoS One. 2023 Feb 15;18(2):e0281056. doi: 10.1371/journal.pone.0281056 (PMC9931102; doi:10.1371/journal.pone.0281056)
Supplement: S1 Table — (DOCX) [file pone.0281056.s015.docx]

**Table S1. Measures of cluster partitions quality for different numbers of clusters**

|  | PBC | HG | HGSD | ASW | ASWw | CH | R2 | CHsq | R2sq | HC |
| --- | --- | --- | --- | --- | --- | --- | --- | --- | --- | --- |
| 2 clusters | 0.50 | 0.75 | 0.75 | 0.71 | 0.71 | 3783.49 | 0.29 | 5076.05 | 0.35 | 0.27 |
| 3 clusters | 0.73 | 0.89 | 0.89 | 0.78 | 0.78 | 4402.10 | 0.49 | 9622.50 | 0.68 | 0.07 |
| 4 clusters | 0.75 | 0.96 | 0.96 | 0.79 | 0.79 | 5857.63 | 0.65 | 12029.88 | 0.80 | 0.02 |
| 5 clusters | 0.69 | 0.96 | 0.96 | 0.81 | 0.81 | 6106.49 | 0.73 | 10387.21 | 0.82 | 0.02 |
| 6 clusters | 0.70 | 0.98 | 0.98 | 0.84 | 0.84 | 6836.60 | 0.79 | 13184.24 | 0.88 | 0.01 |
| 7 clusters | 0.71 | 0.99 | 0.99 | 0.87 | 0.87 | 7148.95 | 0.82 | 14653.68 | 0.90 | 0.01 |
| 8 clusters | 0.70 | 0.98 | 0.98 | 0.84 | 0.84 | 6836.60 | 0.79 | 13184.24 | 0.88 | 0.01 |

PCB: Point Biserial Correlation

HG: Hubert’s Gamma

HGSD: Hubert’s Somers’ D

ASW: Average Silhouette Width

ASWw: Average Silhouette Width (weighted)

CH: Calinski-Harabasz index

R2: Pseudo R²

CHsq: Calinski-Harabasz index squared

R2sq: Pseudo R² squared

HC: Hubert’s C
