# Supplementary material for: Working life sequences over the life course among 9269 women and men in Sweden; a prospective cohort study
Source: PLoS One. 2023 Feb 15;18(2):e0281056. doi: 10.1371/journal.pone.0281056 (PMC9931102; doi:10.1371/journal.pone.0281056)
Supplement: S2 Table — (DOCX) [file pone.0281056.s016.docx]

**Table S2. Transition probabilities between different activity**

|  | Active | On parental leave | Unemployed | On SA/DP* | Retired |
| --- | --- | --- | --- | --- | --- |
| Active | 0.97 | 0.01 | 0.01 | 0.01 | 0.00 |
| On parental leave | 0.83 | 0.21 | 0.03 | 0.01 | 0.00 |
| Unemployed | 0.53 | 0.01 | 0.40 | 0.05 | 0.00 |
| On SA/DP* | 0.27 | 0.01 | 0.04 | 0.67 | 0.01 |
| Retired | 0.09 | 0.00 | 0.00 | 0.00 | 0.91 |

*Sickness absence/disability pension
