# Supplementary material for: Working life sequences over the life course among 9269 women and men in Sweden; a prospective cohort study
Source: PLoS One. 2023 Feb 15;18(2):e0281056. doi: 10.1371/journal.pone.0281056 (PMC9931102; doi:10.1371/journal.pone.0281056)
Supplement: S3 Table — (DOCX) [file pone.0281056.s017.docx]

**Table S3**

**Measures of cluster partitions quality for different numbers of clusters among men**

|  | PBC | HG | HGSD | ASW | ASWw | CH | R2 | CHsq | R2sq | HC |
| --- | --- | --- | --- | --- | --- | --- | --- | --- | --- | --- |
| 2 clusters | 0,894553 | 0,999976 | 0,999976 | 0,955752 | 0,955785 | 3039,287 | 0,390138 | 16482,61 | 0,776251 | 0,000204 |
| 3 clusters | 0,504389 | 0,947253 | 0,947209 | 0,761404 | 0,761613 | 3235,17 | 0,576662 | 9597,115 | 0,801622 | 0,047153 |
| 4 clusters | 0,522599 | 0,970723 | 0,970684 | 0,846427 | 0,846651 | 4766,242 | 0,750679 | 16094,63 | 0,910452 | 0,016227 |
| 5 clusters | 0,47452 | 0,983817 | 0,983769 | 0,877687 | 0,87798 | 5612,866 | 0,825438 | 13647,88 | 0,919986 | 0,007214 |
| 6 clusters | 0,474602 | 0,992685 | 0,992666 | 0,899625 | 0,899974 | 5722,538 | 0,857703 | 12161,97 | 0,92759 | 0,003574 |

**Measures of cluster partitions quality for different numbers of clusters among women**

|  | PBC | HG | HGSD | ASW | ASWw | CH | R2 | CHsq | R2sq | HC |
| --- | --- | --- | --- | --- | --- | --- | --- | --- | --- | --- |
| 3 clusters | 0,524392 | 0,769093 | 0,768755 | 0,559305 | 0,559554 | 1754,709 | 0,437451 | 2875,903 | 0,560342 | 0,120383 |
| 4 clusters | 0,623228 | 0,917835 | 0,917472 | 0,711773 | 0,712126 | 2572,485 | 0,631055 | 5958,195 | 0,798451 | 0,028143 |
| 5 clusters | 0,569203 | 0,915209 | 0,914757 | 0,727523 | 0,727979 | 2596,248 | 0,697167 | 5073,484 | 0,818141 | 0,031963 |
| 6 clusters | 0,573056 | 0,948459 | 0,948318 | 0,777122 | 0,777585 | 2767,135 | 0,754166 | 4994,89 | 0,847038 | 0,020587 |

PCB: Point Biserial Correlation

HG: Hubert’s Gamma

HGSD: Hubert’s Somers’ D

ASW: Average Silhouette Width

ASWw: Average Silhouette Width (weighted)

CH: Calinski-Harabasz index

R2: Pseudo R²

CHsq: Calinski-Harabasz index squared

R2sq: Pseudo R² squared;

HC: Hubert’s C
