# Supplementary material for: Working life sequences over the life course among 9269 women and men in Sweden; a prospective cohort study
Source: PLoS One. 2023 Feb 15;18(2):e0281056. doi: 10.1371/journal.pone.0281056 (PMC9931102; doi:10.1371/journal.pone.0281056)
Supplement: S4 Table — (DOCX) [file pone.0281056.s018.docx]

**Table S4. Multinomial regression analysis for the associations between sex and different working life sequence clusters for alternative 6 clusters solutions**

| Cluster number ordered by numbers of individuals | Number of individuals | Base model  OR (95% CI) for women compared with men | Multi-adjusted model  OR (95%CI) for women compared with men |
| --- | --- | --- | --- |
| 4 clusters solution |  |  |  |
| 1 | 6744 | Ref | Ref |
| 2 | 1179 | 32.0(25.0, 41.1) | 31.4(24.3, 40.7) |
| 3 | 1018 | 1.0(0.9, 1.2) | 1.0 (0.8, 1.1) |
| 4 | 328 | 0.9(0.7, 1.1) | 0.8(0.6, 1.0) |
| 6 clusters solution |  |  |  |
| 1 | 6034 | Ref. | Ref. |
| 2 | 1018 | 1.1 (1.0,1.3) | 1.0(0.9,1.2) |
| 3 | 847 | 23.9 (18.5,30.8) | 22.6 (17.3, 29.6) |
| 4 | 710 | 1.8 (1.5, 2.1) | 1.8(1.4, 2.1) |
| 5 | 332 | 222.9(82.6, 601.6) | 184.0(76.3, 443.8) |
| 6 | 328 | 1.0(0.8,1.2) | 0.9(0.7,1.1) |

OR: odds ratio; CI: confidence interval;

Base model: adjusted for age.

Multi-adjusted model: adjusted for sociodemographic and socioeconomic: age, education, both parents/participants themselves was born outside Sweden, nationality, family situation, living area, economic hardship; health-related factors: previous sickness absence/ disability pension, long-term illness or health-problem, daily smoking and overweight/obesity; and work-related factors: working hours, physically strenuous work, monotonicity, noise exposure, mentally strenuous job, hectic work schedule, opportunity to learn new things and job accidents.
