# Supplementary material for: Working life sequences over the life course among 9269 women and men in Sweden; a prospective cohort study
Source: PLoS One. 2023 Feb 15;18(2):e0281056. doi: 10.1371/journal.pone.0281056 (PMC9931102; doi:10.1371/journal.pone.0281056)
Supplement: S5 Table — (DOCX) [file pone.0281056.s019.docx]

|  | Odds ratios (OR) and 95% confidence intervals (CI) for the membership of activity sequence clusters (ref.: active, n=3601) | | | |
| --- | --- | --- | --- | --- |
|  | Unemployment & SA/DP periods (n=593) | Parental-leave periods (n=77) | SA/DP periods (n=300) | Retirement  (n=182) |
|  | OR (95% CI) | OR (95% CI) | OR (95% CI) | OR (95% CI) |
| Age (ref<31 years) |  |  |  |  |
| 31-40 | 1.2 (0.9, 1.6) | 0.7 (0.4, 1.3) | 1.6 (1.1, 2.5) | 1.7 (0.9, 3.2) |
| 41- | 1.4 (1.0, 1.8) | 0.1 (0.0, 0.4) | 2.7 (1.8.4.1) | 7.8 (4.5, 13.7) |
| Education (ref: compulsory) |  |  |  |  |
| Secondary | 1.0 (0.7, 1.2) | 0.7 (0.3, 1.4) | 0.7 (0.5, 0.9) | 0.6 (0.4, 0.9) |
| University, college | 0.6 (0.4, 0.8) | 0.9 (0.4, 2.0) | 0.5 (0.3, 0.7) | 0.7 (0.4, 1.1) |
| Type of living area (ref: Stockholm) |  |  |  |  |
| Gothenburg/ Malmö | 1.3 (0.9, 1.7) | 0.7 (0.4, 1.4) | 1.5 (1.1, 2.5) | 1.1 (0.6, 1.8) |
| Other larger cities | 1.4 (1.1, 1.9) | 0.5 (0.3, 0.9) | 0.8 (0.6, 1.2) | 0.8 (0.5, 1,2) |
| Small, middle size towns, rural | 1.5 (1.1, 2.0) | 0.6 (0.3, 2.2) | 0.9 (0.6, 1.3) | 1.1 (0.6, 1.7) |
| Family situation (ref: living alone without children) | | | | |
| Cohabiting with children | 0.6 (0.4, 0.7) | 0.9 (0.4, 1.7) | 0.8 (0.5, 1.1) | 0.3 (0.2, 0.5) |
| Cohabiting without children | 0.6 (0.5, 0,8) | 2.0 (1.0, 3.5) | 0.8 (0.5, 1.2) | 0.6 (0.4, 0.9) |
| Living alone with children | 0.4 (0.2, 0.7) | 2.0 (0.5, 7.2) | 0.6 (0.3, 1.2) | 0.4 (0.2, 0.8) |
| Not Swedish citizen (ref: Swedish) | 1.3 (0.9, 2.0) | 0.4 (0.1,3.7) | 1.7 (0.9, 3.4) | 1.5 (0.7, 3.2) |
| Both parents/themselves born outside Sweden (ref: at least one parent born in Sweden) | 2.2 (1.7, 2.9) | 0.8 (0.3, 2.0) | 1.1 (0.7. 1.7) | 1.2 (0.7, 2.1) |
| Economic hardship (ref: no) | 2.0 (1.6, 2.5) | 1.0 (0.5, 3.9) | 1.2 (0.9, 1.7) | 1.1 (0.7, 1.7) |
| Health-related variables |  |  |  |  |
| Daily smoker | 1.8 (1.4, 2.6) | 1.1 (0.5, 2.3) | 1.6 (1.2, 2.2) | 2.2 (1.5, 3.1) |
| Overweight/obese (BMI> 25 kg/m²) | 1.1 (0.9, 1.3) | 1.0 (0.6, 1.6) | 1.6 (1.2, 2.0) | 1.4 (1.0, 1.9) |
| SA/DP during the previous year | 5.6 (2.6, 12.0) | 4.1 (0.6, 33.0) | 15.8 (7.9, 31.6) | 2.3 (0.6, 8.6) |
| Long-term illness or health problem | 1.3 (1.0, 1.5) | 1.3 (0.8, 2.2) | 2.6 (2.0, 3.4) | 1.8 (1.3, 2.5) |
| Poor self-rated health | 2.9 (1.7, 5.1) | 1.1 (0.1,8.2) | 5.4 (3.1, 9.6) | 3.2 (1.4, 7.3) |
| Work-related variables |  |  |  |  |
| Weekly working hours (ref: >35 and <45 hours) | | | | |
| ≥45 hours | 0.9 (0.6, 1.1) | 1.1 (0.6, 2.1) | 1.2 (0.9, 1.7) | 1.1 (0.7, 1.6) |
| ≤35 hours | 1.3 (1.0, 1.8) | 1.1 (0.5, 2.3) | 1.3 (0.9, 2.1) | 1.3 (0.7, 2.3) |
| Mentally strenuous job | 1.1 (0.9, 1.3) | 1.9 (0.5, 1.5) | 1.1 (0.8, 1.5) | 1.2 (0.8, 1.6) |
| Had workplace accident | 1.2 (0.9, 1.6) | 0.9 (0.4, 2.1) | 0.8 (0.6, 1.3) | 1.3 (0.8, 2.2) |
| Hectic schedule | 0.8 (0.6, 0.9) | 1.2 (0.7, 2.0) | 1.1 (0.8, 1.5) | 0.9 (0.6, 1.2) |
| Little/no opportunity to learn new things | 0.8 (0.6, 1.0) | 0.7 (0.4, 1.2) | 0.9 (0.7, 1.2) | 0.9 (0.6, 1.3) |
| Monotonous job | 1.4 (1.2, 1.8) | 1.4 (0.9, 2.3) | 1.1 (0.8, 1.4) | 1.5 (1.0, 2.1) |
| Physically strenuous job | 1.0 (0.8, 1.3) | 1.0 (0.5, 1.9) | 1.4 (1.0, 1.0) | 0.9 (0.6, 1.3) |
| Exposed to noise | 0.7 (0.6, 0.9) | 0.5 (0.3, 0.9) | 0.9 (0.7, 1.3) | 0.8 (0.5, 1.1) |

**Table S5. Associations between baseline factors and cluster membership among women** SA/DP: sickness absence/disability pension; BMI: body mass index
