# Supplementary material for: Correction: Working life sequences over the life course among 9269 women and men in Sweden; A prospective cohort study
Source: PLoS One. 2025 Mar 5;20(3):e0319833. doi: 10.1371/journal.pone.0319833 (PMC11882063; doi:10.1371/journal.pone.0319833)
Supplement: S5 Table — (DOCX) [file pone.0319833.s001.docx]

|  | Odds ratios (OR) and 95% confidence intervals (CI) for the membership of activity sequence clusters (ref.: active, n=2433) | | | |
| --- | --- | --- | --- | --- |
|  | Unemployment & SA/DP periods (n=425) | Parental-leave periods (n=1102) | SA/DP periods (n=410) | Retirement  (n=146) |
|  | *OR (95% CI)* | *OR (95% CI)* | *OR (95% CI)* | *OR (95% CI)* |
| Age (ref<31 years) |  |  |  |  |
| 31-40 years | 0.9 (0.6, 1.2) | 0.25 (0.2, 0.3) | 1.5 (1.0, 2.3) | 0.4 (0.2 ,0.9) |
| 41- years | 0.6 (0.4, .08) | 0.0 (0.0, 0.0) | 1.5 (1.0, 2.3) | 2.0 (1.2, 3.6) |
| Education (ref: compulsory) |  |  |  |  |
| Secondary | 0.7 (0.5, 1.0) | 1.4 (1.0, 2.0) | 0.7 (0.5, 1.0) | 1.0 (0.5, 1.7) |
| University, college | 0.4 (0.3, 0.6) | 1.7 (1.1, 2.4) | 0.6 (0.4, 0.8) | 1.1 (0.6, 2.0) |
| Type of living area (ref: Stockholm) |  |  |  |  |
| Gothenburg/Malmö | 1.5 (1.0, 2.2) | 1.0 (0.8, 1.4) | 1.4 (1.0, 2.1) | 1.3 (0.7, 2.2) |
| Other larger cities | 1.7 (1.2, 2.9) | 0.9 (0.7, 1.1) | 1.6 (1.1, 2.2) | 0.9 (0.6, 1.5) |
| Small, middle size towns, rural | 1.5 (1.0, 2.1) | 1.0 (0.7, 1.2) | 1.4 (1.0, 2.1) | 0.9 (0.6, 1.6) |
| Family situation (ref: living alone without children) | | | | |
| Cohabiting with children | 0.7 (0.5, 1.0) | 1.2 (0.9, 1.5) | 0.9 (0.6, 1.3) | 0.4 (0.2, 0.6) |
| Cohabiting without children | 0.6 (0.4, 0.9) | 1.3 (1.0, 1.7) | 0.9 (0.6, 1.4) | 1.6 (0.9, 2.6) |
| Living alone with children | 0.9 (0.6, 0.9) | 1.0 (0.7, 1.5) | 1.6 (1.0, 2.5) | 0.5 (0.2, 1.0) |
| Not Swedish citizen (ref: Swedish citizen) | 0.9 (0.6, 1.4) | 1.2 (0.9, 1.7) | 0.8 (0.4, 1.4) | 1.3 (0.6, 3.1) |
| Both parents/themselves being born outside Sweden (ref: born in Sweden or at least one parent born in Sweden) | 2.1 (1.5, 2.9) | 1.0 (0.6, 1.6) | 1.6 (1.1, 2.3) | 1.4 (0.8, 2.5) |
| Economic hardship (ref: no) | 2.0 (1.5, 2.6) | 1.4 (1.1, 1.8) | 0.9 (0.6, 1.2) | 1.1 (0.6, 2.0) |
| Health-related factors |  |  |  |  |
| Daily smoker | 1.4 (1.1, 1.9) | 1.0 (0.8, 1.3) | 1.4 (1.0, 1.8) | 1.2 (0.8, 1.8) |
| Overweight/obese (BMI>25 kg/m²) | 1.2 (0.9, 1.5) | 1.3 (1.1, 1.6) | 1.4 (1.1, 1.8) | 1.0 (0.7, 1.5) |
| SA/DP during the previous year | 5.5 (2.6, 11.7) | 4.1 (1.7, 9.8) | 17.6 (9.2, 33.9) | 9.1 (3.5, 23.6) |
| Long-term illness or health problem | 1.9 (1.5, 2.4) | 0.9 (0.8, 1.1) | 2.8 (1.6, 4.9) | 1.2 (0.8, 1.7) |
| Poor self-rated health | 2.5 (1.4, 4.4) | 1.6 (0.8, 3.2) | 3.9 (1.6, 9.5) | 0.6 (0.1, 2.5) |
| Work-related variables |  |  |  |  |
| ..Weekly working hours (ref: 35 and <45 hours) | | | | |
| ≥45 hours | 1.1 (0.7, 1.9) | 1.0 (0.7, 1.2) | 0.9 (0.5, 1.5) | 0.8 (0.4, 1.7) |
| ≤35 hours | 1.2 (0.9, 1.5) | 0.7 (0.6, 0.8) | 1.1 (0.8, 1.3) | 1.4 (1.0, 2.1) |
| Mentally strenuous job | 0.8 (0.6, 1.0) | 1.1 (0.9, 1.3) | 1.2 (1.0, 1.6) | 1.0 (0.7, 1.4) |
| Had workplace accident | 1.0 (0.6, 1.0) | 1.0 (0.7, 1.4) | 1.3 (0.8, 1.9) | 1.3 (0.7, 2.5) |
| Hectic schedule | 1.0 (0.8, 1.3) | 1.2 (1.0, 1.5) | 1.2 (0.9, 1.6) | 1.0 (0.6, 1.5) |
| Little/no opportunity to learn new things | 0.8 (0.6, 1.0) | 1.2 (1.0, 1.4) | 0.9 (0.7, 1.2) | 0.9 (0.6, 1.3) |
| Monotonous job | 1.1 (0.9, 1.4) | 1.0 (0.8, 1.3) | 1.2 (0.9, 1.6) | 1.2 (0.8, 1.7) |
| Physically strenuous job | 0.8 (0.6, 1.1) | 0.9 (0.7, 1.2) | 0.9 (0.7, 1.3) | 0.8 (0.5, 1.3) |
| Exposed to noise | 1.1 (0.8, 1.4) | 0.8 (0.7, 1.1) | 0.9 (0.7, 1.2) | 1.1 (0.7, 1.7) |

**Table S5.** **Associations between predictors and cluster membership among women**
